# The Comeback of the Old Theological Narratives During the Coronavirus Crisis: A Critical Reflection

**DOI:** 10.1007/978-3-030-65355-2_19

**Published:** 2021-03-20

**Authors:** Jan Loffeld

**Affiliations:** 1 Tilburg University, Tilburg, The Netherlands; 2 Tilburg University, Tilburg, The Netherlands; 3 Tilburg University, Tilburg, The Netherlands; 4 Tilburg University, Tilburg, The Netherlands; Department of Practical Theology and Religious Studies, Tilburg School of Catholic Theology, Tilburg, The Netherlands

## Abstract

Martin Luther had no doubt about it: diseases were a punishment from God. In espousing this view, Luther, who was one of the first people to translate the Bible from Greek into another language, stood on firm biblical grounds. For the Semitic people of the biblical world, this causal connection had been self-evident as well. Diseases, plagues, catastrophes were the consequences of the sin that people commit. Ultimately, the intuition that evil is the result of sin is the basis for the adage that adversity causes people to pray: sooner or later, human beings will be confronted with the contingency of their own lives, which, in the Christian perspective, is rooted in the fact that creation has fallen into sin. This is why the idea that adversity causes people to pray is often trotted out in times of crisis even though it has long been empirically disproven.

Martin Luther had no doubt about it: diseases were a punishment from God. In espousing this view, Luther, who was one of the first people to translate the Bible from Greek into another language, stood on firm biblical grounds. For the Semitic people of the biblical world, this causal connection had been self-evident as well. Diseases, plagues, catastrophes were the consequences of the sin that people commit. Human beings themselves—Adam and Eve—introduced evil into creation, which was good in itself before, and they must bear the consequences. According to the doctrine of original sin, as it was later refined after this so-called “Fall of Man,” human beings are even born into a primeval state of guilt that is propagated from generation to generation, and from which they cannot liberate themselves. For Paul, in his letter to the Romans, this notion underlay the redemption, which he envisaged as being universal: sin is all-encompassing and so is the liberation from servitude that God works in Jesus Christ. Ultimately, the intuition that evil is the result of sin is the basis for the adage that adversity causes people to pray: sooner or later, human beings will be confronted with the contingency of their own lives, which, in the Christian perspective, is rooted in the fact that creation has fallen into sin. This is why the idea that adversity causes people to pray is often trotted out in times of crisis even though it has long been empirically disproven (Heuft 2017).

## The Nature of Redemption

The question of the nature of redemption was so important around the time of the Reformation that it sparked off a global schism. Salvation or damnation, being included among or excluded from the elect, and the distinction between “good and evil”—these have long been important questions in the history of Christianity. Religion served to bring clarity and certainty to these questions. Ultimately, what was at stake was the question how religion gives meaning to the fact that human life escapes control or manipulation, i.e., is “unavailable” and contingent.

But there are at least two things that cast doubt upon the clarity offered by religion. First of all, there is a biblical issue: Jesus tells his disciples the story of eighteen people who died when a tower collapsed upon them and then provocatively asks them, “Do you suppose that these Galileans were worse sinners than any others?” (Lk 13: 4). Secondly, the Lisbon earthquake of the eighteenth century was the event that gave rise, historically, to the theodicy, which questioned beliefs hitherto regarded as plausible. Thousands of innocent people perished gruesomely due to a seemingly interminable city fire; only the red-light district escaped. Here, finally, the causality of a God who punishes justly, who rewards the good and damns the wicked floundered. There was empirical evidence here that the Christian causal link between the human act and historical occurrence was epistemically flawed.

## The Mystery of Evil

In modernity, this was one of the reasons for the emergence of a new theological model of thinking. Human beings have become emancipated before God, yet they remain prone to guilt, and phenomenologically, guilt has not disappeared from the world. It still requires an explanation that can also function without reference to God. Karl Rahner (1984) articulated this in his Foundations of Christian Faith as “the fact that people are always under threat from themselves.” To use a classical expression: human beings, in one way or another, become wolves to themselves: if we accept the idea of God, this is because original sin is still at work in them; if we do not, it is because “radical evil” (Kant 1792) cannot be explained or eradicated even with the use of reason. Rahner (1984) called this the “mystery of evil.” Whereas previously, evil in the form of epidemics or natural catastrophe could be explained as resulting from the fact that human beings—creation in its entirety—had brought original sin upon themselves by sinning against God, now, human beings themselves, without explicit reference to God, have become the authors of punishment for wrongdoing. Other factors are personified: “Nature strikes back!” or “Climate change is the way nature takes revenge for exploitative modern life.” The theodicy has been transformed into an anthropodicy: the question why people can be the cause of evil.

It is, or was, interesting to see during the coronavirus crisis how these old interpretative patterns or narratives returned as human beings were confronted with the hitherto unimagined intrusion of human vulnerability or contingency. To give just two examples: the Dutch newspaper Reformatorisch Dagblad wrote in early March 2020 that the expansion of the coronavirus threat into a true pandemic was due to “the government of God”: “Christians confess that illness and health do not happen to them by coincidence. […] The coronavirus is only a secondary cause, a means in the hands of God. He sends disease like once He sent the plagues to Egypt to bring humankind to repentance.”^1^

Almost at the same time, the Brazilian liberation theologian Leonardo Boff published an article entitled “The Origins of the Coronavirus.” The opening words were: “From this moment on we have everything to fear, even the destruction of the human race; this would be the just price for our foolishness and barbarity.” This idea is premised upon the “Gaia hypothesis” which represents “Mother Earth” as a “self-regulating superorganism, as a living being, that is able to feel, think, love and care for itself.”^2^ It is no longer God but the Earth who punishes. Both interpretations thus use personifications as the metaphor, and both, in equal measure, believe that the guilt lies with human beings, who receive just punishment.

The theological legitimacy of both interpretations has been and still is subject to heated debate. However, it is important to ask a different question here: does an old “grand narrative”—that is what Christianity undoubtedly is in Europe—really do itself any favors in an increasingly secularizing society by using an old “little narrative” to explain and give meaning to human life? To put it differently: does Christianity truly do justice to its fundamental mission to speak of the God of Life by advancing such interpretations?

This question arises from the underlying impression that the return of these old narratives, specifically in times of crisis, is primarily fed by resentment. The Indian cultural scholar Pankaj Mishra has recently pointed out in a profound analysis how strongly human actions in general, as well as developments in cultural history, spring from a feeling of resentment, i.e., the specific feeling of having been short-changed:

An existential resentment concerning the being of other people, provoked by an intense mixture of envy and the feeling of humiliation and impotence; a resentment that is always there and is ever increasing, that is poisoning civil society and undermining political freedom, and that is currently bringing about a turn to authoritarianism and dangerous forms of chauvinism across the globe (Mishra 2017: 25)*.*

This argument, which Mishra develops very broadly and highly plausibly, can also be applied to aspects of current religious life. For Christians, it can lead to the belief that Christianity’s deepest wound in modernity is its loss of the monopoly on interpreting human life and its fate. It continues to be a challenge for Christians to truly respect the autonomy of the world, and at the same time, to believe that the world has not been abandoned to its own fate. When the old narratives are repeated, it is often forgotten how dark the image of God is (a punishing God, or a God who deistically abandons creation to its own fate) and how crude the anthropology that these old narratives convey. Moreover, the notion that adversity teaches people to pray is based upon a functional understanding of religion, which runs the risk of turning God into an idol for personal desires and needs, only to be jettisoned again when it is no longer required.

## Human Unavailability

An alternative for these resentment-based coping strategies is an understanding of Christianity as an option for interpreting the world. This option is then also presented as such in the market of worldviews. The German sociologist Hartmut Rosa (2019) has shown in his latest book Unverfügbarkeit (Unavailability) how this can be done in a way that could appeal to contemporary culture. This volume, published in 2019, directly engages those experiences of vulnerability that modern societies going through the coronavirus crisis must existentially process. Rosa regards the attempt to render the world universally “available” through modernity as failed and argues by contrast for the “acceptance of the unavailable.”

It is perhaps no coincidence that the concept of unavailability originally derives from a theological context. Theology uses it to express a foundational element of the human relationship to the world, which continues to be of sociological and philosophical, as well as psychological interest, even if all theological or metaphysical assumptions about the essence of God (or even about whether there is any essence of God at all) are rejected. […] In my lay opinion, the core of the Jewish-Christian image of God consists entirely of a resonance-theoretical representation: even if God […] is thought as fundamentally unavailable, the relationship between God and humankind is nevertheless conceived as one of mutual accessibility-for-relatedness: […] responsivity here means […], so to speak, a hearing, attentive interrelatedness, which has a transformative power, but at the same time respects both sides’ ‘own voice’ and ability to answer: whether resonance arises, and what its outcome is going to be remains unavailably undetermined. In my view this conception underlies the practice of prayer […] (Rosa, 67f.)*.*

This alternative interpretation of contingency or human unavailability, which even modernity cannot wipe out, piques our theological attention specifically during the coronavirus crisis. Christianity has an option to offer: a God whose image has been liberated from the constraints of functionality and from a dark anthropology and theology; an option that can help to process human unavailability—but without any coercion and freed from universal claims (such as that the world has fallen in sin) that are, in any case, no longer plausible in secular cultures. Christian theology can reflect on what it means to see the world etsi Deus daretur (“as if there were a God”), and pastoral ministry can offer the related practice. But this must not be communicated to all people by any possible means, to avoid overwhelming their free will. It will free us from feelings of superiority (including those of a fundamentalist nature) and will possibly give rise to new pastoral creativity, which has in fact already been evident during the coronavirus crisis. Therefore, this way of dealing with human contingency can during times of crisis as well as during ordinary times, do greater justice to both Christian self-understanding and the just principles of liberty that rule modern societies than the reassertion of older, often resentment-based narratives can. It would be appropriate if this were also to become the “new common” in theological reflection and teaching.

## Footnotes

1 Cf. on this: https://www.rd.nl/opinie/commentaar/niet-corona-heerst-maar-god-regeert-1.1638981 (10 June 2020).

2 Cf. on this: https://www.tagesanzeiger.ch/leben/gesellschaft/der-gerechte-preis-fuer-unsere-grausamkeit/story/29234175 (10 June 2020).
